# The prospective application of a graphene/MoS_2_ heterostructure in Si-HIT solar cells for higher efficiency†

**DOI:** 10.1039/d0na00309c

**Published:** 2020-06-23

**Authors:** Chandra Kamal Borah, Pawan K. Tyagi, Sanjeev Kumar

**Affiliations:** Centre of Advanced Research, Department of Physics, Rajiv Gandhi University Arunachal Pradesh-791112 India sanjeev.kumar@rgu.ac.in; Department of Physics, Central University of Haryana Haryana-123029 India; Department of Applied Physics, Delhi Technological University Delhi-110042 India

## Abstract

The efficiency of a Si-HIT (heterojunction with intrinsic thin layer) solar cell based on a graphene/MoS_2_ heterostructure has been optimized by varying the various parameters of graphene (Gr) as a transparent conducting electrode (TCE) and n-type molybdenum disulfide (n-MoS_2_) as an emitter layer. The photovoltaic performance of a graphene/n-MoS_2_/a-Si:H/p-cSi/Au single facial HIT solar cell has been studied using AFORS-HET v2.5 simulation software. A maximum output efficiency of 25.61% has been achieved. The obtained results were compared with the results from a commercially available a-Si:H layer and p-cSi wafer after simulation. Moreover, the dependence of the cell performance on changes in the TCE and the back contact materials has also been studied. Finally, it has been demonstrated that the graphene layer and n-MoS_2_ layer could act as a TCE and an efficient emitter layer, respectively, in a n-MoS_2_/p-cSi based HIT solar cell.

## Abstract 2

Graphene, MoS_2_ and silicon-based HIT solar cell with 25.61% output efficiency.

## Graphical abstract

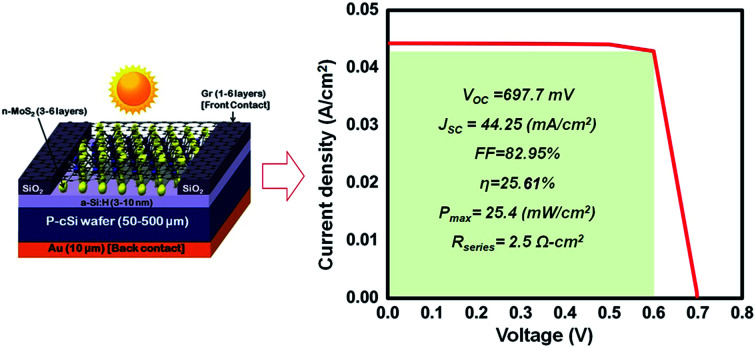

## 1. Introduction

The output efficiency (*η*) of a simple silicon (Si) heterojunction solar cell degrades owing to the presence of a large number of defects at the surface of the Si wafer. This deficiency of heterojunction solar cells can be overcome by the introduction of a thin intrinsic layer between the two doped layers of the heterojunction. Such a structure is known as a heterojunction with intrinsic thin layer (HIT) structure.^1^ In a HIT structured solar cell, the intrinsic layer-like a-Si:H reduces the interfacial recombination state density to the minimum level between two doped layers^2^ and reduces the leakage current.^3^ Recently, SANYO Ltd reported attaining a 20% output efficiency in HIT solar cells based on n-type Si.^4^ Furthermore, Panasonic has achieved the world's highest efficiency of 25.6% in a HIT cell based on an n-type c-Si.^5^

In a solar cell, a transparent conductive electrode (TCE) is required to ensure the easy transportation of carriers from the emitter layer to the external circuit. The low sheet resistance (10 Ω sq^−1^) and high optical transparency (85%) mean that indium tin oxide (ITO) is a commonly used TCE in photovoltaic (PV) applications. However, the high cost, rarity and mechanical rigidity of ITO have limited its use for future flexible devices and these drawbacks initiated the search for new TCE materials.^6^ Recently, pristine graphene has been reported to have an optical transparency as high as 97.7% (single layer graphene) with a work function of 4.31–4.5 eV (ref. 7 and 8) and a sheet resistance of 120 Ω sq^−1^ in few-layer graphene.^6^ In addition, the intercalated graphene with ferric chloride is reported to have a high optical transparency of approximately 96% with a low sheet resistance of 8.8 Ω sq^−1^ and a high carrier density of 8.9 × 10^14^ cm^−2^ at room temperature and a high stability in air.^6^ At room temperature, the carrier mobility in the graphene layer exceeds 10^5^ cm^2^ V^−1^ s^−1^.^9^ These extraordinary properties have led to the application of multilayer graphene as a TCE. Recently, Lazar *et al.*^7^ reported that doping with boron (B) and –NH_2_ can increase the work function of graphene (*Φ*_Gr_) to 1.20 eV and 0.46 eV from 4.31 eV (pristine graphene). Kim *et al.*^8^ demonstrated that, after being irradiated by an *α* beam the work function of graphene increases to 4.9 eV. Conversely, the low photoresponsivity, poor external quantum efficiency, and low absorption of light reduces the application of graphene as an emitter layer instead of the TCE layer in HIT solar cell.^10^ Unlike graphene, another 2D material, molybdenum disulfide (MoS_2_) has emerged as a complement 2D material for graphene as an emitter layer in HIT solar cells. MoS_2_ has a mobility of 200 cm^2^ V^−1^ s^−1^ in the monolayer and approximately 517 cm^2^ V^−1^ s^−1^ in few layers, as well as a high on/off ratio in the order of 10^8^ (ref. 11 and 12) and a fast photo-response time of 40 μs.^13^ MoS_2_ is reported to have an indirect bandgap of 1.2 eV in the bulk form and a direct bandgap of 1.9 eV in the single layer form.^14^ Moreover, double layer MoS_2_ has more than 85% optical transparency in the visible region.^15^ Recently, it has been reported that the work function of MoS_2_ changes with a change in the number of layers.^16^ It has also been demonstrated that MoS_2_ can be used to form significant n-type doping with a Benzyl Viologen (BV) dopant^17^ and polyethyleneimine (PEI)^18^. PEI reduces the sheet resistance of MoS_2_ 2.6 times.^18^ These properties mean that MoS_2_ can be utilized as an emitter layer in MoS_2_/silicon HIT solar cells.

Experimentally and theoretically, it has been demonstrated that MoS_2_ can act as an emitter layer in heterojunction silicon solar cells. Tsai *et al.*^19^ fabricated a heterojunction solar cell of monolayer MoS_2_ and p-cSi and achieved a power conversion efficiency of 5.23%. Hao *et al.*^20^ also fabricated a bulk p–n junction after depositing a 40 nm thick layer of n-MoS_2_ onto p-cSi substrates and a 1.3% photoconversion efficiency was achieved.

The high transparency, as well as the high mobility of the graphene layer with a tunable bandgap and work function, along with practical and theoretical demonstrations of graphene/MoS_2_ and MoS_2_/Si heterojunctions, motivated us to propose a single facial HIT solar cell structure of graphene/n-MoS_2_/a-Si:H/p-cSi/Au. Here, we have used graphene as a TCE material. No reports have been published on the use of graphene as a TCE in HIT solar cells so far. In addition to graphene and MoS_2_, there are a few interesting candidates such as carbon nanotubes, black phosphorous and MXene, that show potential for use in silicon solar cells. So far, a power conversion as high as 17.2% has been observed in carbon nanotube based silicon solar cells.^21–23^ After proposing the structure of the cell, the influence of various parameters of the layers used on the cell performance were studied using AFORS-HET v2.5 simulation software. The main objective of the present simulation is to study the independent effects owing to the variation of the parameters of the layers on the cell performance and to demonstrate the prospective application of graphene as an effective TCE material in a n-MoS_2_/p-cSi HIT solar cell.

## 2. Physical model, structural model and formation of the junctions

### 2.1 Physical model

AFORS-HET is solar cell simulation software, used to study and analyze the various properties of a heterojunction solar cell. For the proposed structure, this software solves Poisson's equation and the transport equation for electrons and holes in one dimension. These equations are as follows:
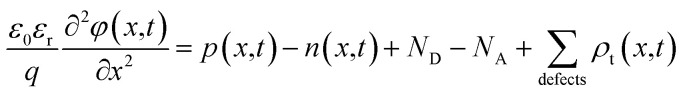

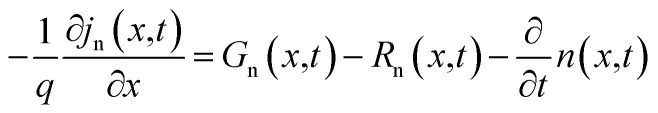

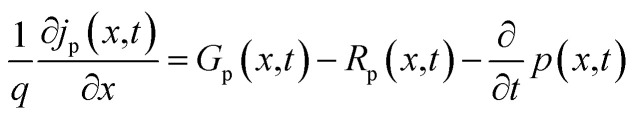
In which, *n* represents the electron density, *p* is the hole density, *φ* is the electric potential, *q* is the electron charge, *ε*_0_*ε*_r_ is the absolute/relative dielectric constant, and *N*_D/A_ represents the donors/acceptors concentrations, which are assumed to be completely ionized. Here, *n*, *p* and *φ* are the independent variables for which the system of differential equations is solved.

The charge stored in the defects is described by a distribution function, *f*_t_, specifying the probability that defects with a defect density *N*_t_ are at position *E* if the bandgap is occupied with electrons:
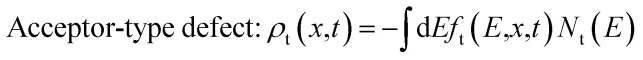

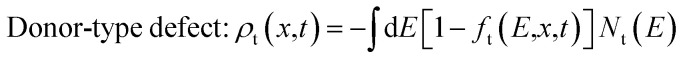


The electron/hole currents (*j*_n/p_) flow owing to the gradient of the corresponding quasi-Fermi energy (*E*_F_n/p__). Within the semiconducting layer, this is equivalent to the sum of the diffusion and drift current with the corresponding mobility *μ*_n/p_.
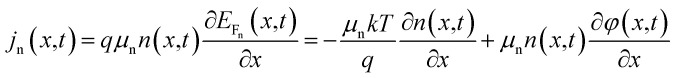

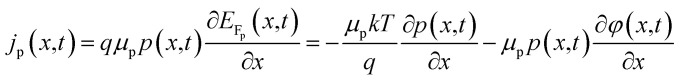


If the heterostructure is illuminated (specifying the spectral distribution *ϕ*(*λ*) of the incoming photon flux), the super-bandgap optical generation rate 
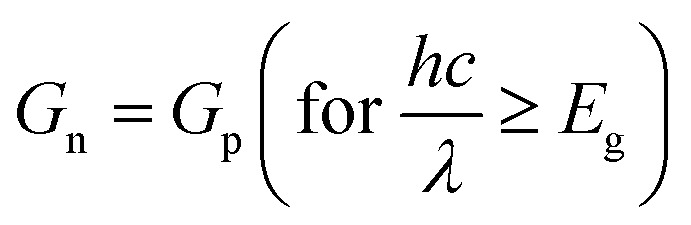
 from the valence band into the conduction band of the semiconductor layers can be evaluated by considering the Lambert–Beer absorption model by specifying the spectral absorption coefficient *α*, as well as specifying the dielectric properties (*n*,*k*) of each layer. An optical sub-bandgap generation 
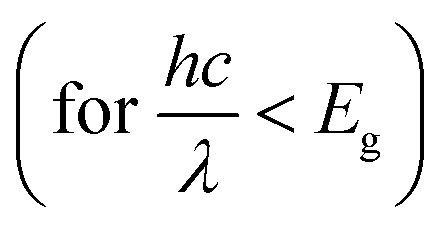
 from a defect to the conduction/valence band can be defined by specifying the optical emission coefficients *e*^o^_n_(*E*),*e*^o^_p_(*E*) ≠ 0 for the defect state.

Recombination from the conduction band into the valence band may occur directly (band to band recombination, Auger recombination) and *via* trap states (Shockley–Read–Hall recombination, (SHR)).*R*_n,p_(*x*,*t*) = *R*^Band–Band^_n,p_(*x*,*t*) + *R*^Auger^_n,p_(*x*,*t*) + *R*^SRH^_n,p_(*x*,*t*)

The transportation across the heterojunction interface is modeled by drift-diffusion. In order to do so, an interface layer is considered. The specified interface defects are distributed homogeneously within this layer. The cell currents have to be evaluated directly from the gradient of the corresponding quasi-Fermi energies. The electron affinity (*χ*), the bandgap (*E*_g_) and the effective conduction/valence band density of states (*N*_C_, *N*_V_) will depend on the position *x* within the interface layer.
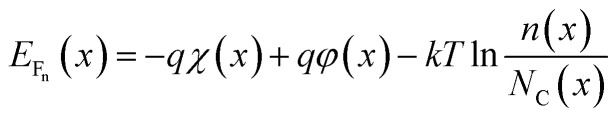

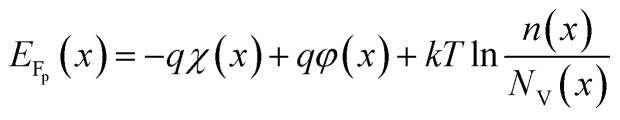


The electric potential is fixed to zero at one contact. At the second contact, a boundary condition has to be specified, which relates the external cell voltage/current to the internal quantities. Furthermore, the electron and hole currents moving into the metal contacts need to be modeled. The Schottky contact can either be voltage controlled, or current controlled.

### 2.2. Structural model

**Fig. 1:**
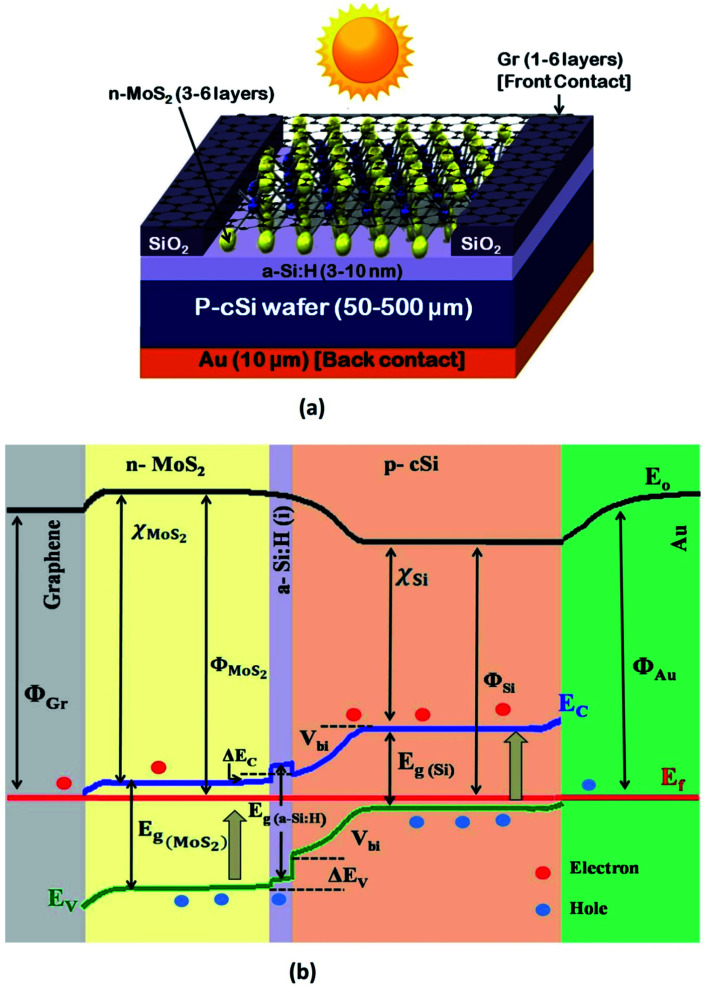
(a) The proposed HIT solar cell structure, and (b) band diagram as generated using AFORS-HET software for the proposed structure.

Layered structure MoS_2_ can be directly deposited on Si substrates. However, owing to their structural mismatch (*i.e.* layered structure of MoS_2_ and diamond-like structure of Si), large quantities of lattice defects are produced and this increases the recombination at the interface. Thus, the introduction of an intrinsic (i) buffer layer a-Si:H could be beneficial to balance the carrier injection and reduce the leakage current in the cell.^3^ Therefore, the as-synthesized MoS_2_ is an n-type semiconductor^24^ and p-type silicon is more popular in the PV industries.^4^ Thus, in this simulation, we have used p-cSi as a base material. Moreover, the minority carrier diffusion length in p-cSi is also reported to be higher than n-cSi.^25^ Furthermore, Fan *et al.*^26^ have reported that ultrathin gold (Au) contacts provide a better electrical conductivity and optical reflectance-scattering to maintain the performance of the cell compared with ITO. Also, it has been demonstrated that an Au/Si interface formed by using novel transfer printing technology makes excellent electrical contact and exhibits a low contact resistance in comparison to that formed by vacuum deposition of Au on Si.^27^ Therefore, the proposed structure of the single facial HIT solar cell is configured as graphene/n-MoS_2_/a-Si:H(i)/p-cSi/Au, as shown in Fig. 1. No lattice mismatch between the layers has been considered for this simulation. Ideally, a semiconductor is called 2D if it satisfies the condition 〈*t*〉 ≤ *λ*_F_, in which 〈*t*〉 is the average system of the 2D electron system, *λ*_F_ is the Fermi wavelength which is equal to
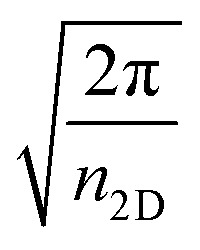
 nm, and *n*_2D_ is the carrier density in cm^−2^.^28^ Following this condition as reported by Sarma *et al.*^29^ and Suh *et al.*^30^ even *n* ∼ 10^5^ cm^−3^ in graphene and *n* ∼ 1.8 × 10^14^ cm^−3^ in MoS_2_ cannot satisfy the above described conditions respectively. It has also been reported that doping in 2D materials increases the interlayer spacing.^31^ Thus, we consider graphene and MoS_2_ quasi 3D in nature instead of 2D. The quantum confinement in the structure was achieved by controlling the layer number of n-MoS_2_. In the proposed HIT structure, as shown in Fig. 1a the carrier transport occurred along the *c*-axis. Details of the front contact and the back contact parameters of the proposed structure are given in Table 1^6,7,26,32–37^ and the parameters, as well as the ranges of the n-MoS_2_, a-Si:H and p-cSi are given in Table 2. The thickness of the single graphene was taken as 0.334 nm and the monolayer MoS_2_ as 0.65 nm.^19,38,39^ However, we started our simulation from five-layer (5L) graphene (1.67 nm) and six-layer (6L) MoS_2_ (3.9 nm) by considering the high overlapping probability of the graphene and p-cSi. It was also reported that the Raman spectrum of 6L MoS_2_ is very close to the bulk^37^ and the optical transparency of 5L graphene (88.5%) is more than that of ITO (85%).^6^ The parameters considered for optimization of the MoS_2_ layer were the donor concentration (*N*_D_), *N*_C_/*N*_V_, *E*_g_, layer thickness, *χ*, dielectric constant (*ε*_r_) and electron mobility (*μ*_n_). The values of these parameters were kept within range and these and the results of the above mentioned previous literature are tabulated in Table 2.^3,40–47^ To simulate and optimize the other parameters the bulk and interfacial defect distribution and recombination rates were kept constant in the software. Furthermore, the MS-Schottky contact model was selected for the graphene/n-MoS_2_ and p-cSi/Au interfaces and the drift-diffusion model was selected for the n-MoS_2_/a-Si:H and a-Si:H/p-cSi interfaces. The values and parameters of these interfaces^48^ are listed in Table 3. The values of these parameters were varied accordingly under air mass 1.5 (AM1.5) illuminations under a power density of 100 mW cm^−2^ and a temperature of 300 K. In order to study the effect of the layer parameters on the cell performance, the open-circuit voltage (*V*_OC_), short circuit current density (*J*_SC_), fill factor (FF) and efficiency (*η*) were analyzed. After optimizing the parameters of n-MoS_2_ further simulation was extended to the a-Si:H layer and the thickness and bandgap were optimized. The values and ranges of these parameters were obtained from previously published studies.^4,49–51^ Further simulations were performed to optimize the parameters, such as the *N*_A_, *N*_C_/*N*_V_, *χ*, and thickness for p-cSi. The values and the ranges of the parameters for the p-cSi layer were taken from the previously published research.^4,47*and*48^ The defect properties were kept as the default. Finally, we optimized the parameters of the contact materials, that is graphene, Au, ITO, Ag, and so forth. The work function (*Φ*) and the number of layers were optimized and other parameters were fixed, as found in the literature.^7,8,34–37,52^

**Table 1:** Front and back contact parameters

Parameter	Front contact			Back contact	
	Initially	After optimization		Initially	After optimization
Material	Graphene	ITO	Al	Au	Ag
Thickness	1L–5L (1L = 0.334 nm)	80 nm	80 nm	10 μm	10 μm
Optical properties	*n* = 2.7	ITO *nk* (default)	*n* = 1.19	Au *nk* (default)	Ag *nk* (default)
	*k* = 1.45		*k* = 7.05		
Work function (eV)	4.31	4.4	4.06	5.4	4.64
	4.50	4.45	4.20		4.74
	4.54	4.5	4.26		
	4.77				
	4.90				
Absorption loss	0.023 (constant)	ITO abs (default)	0.056	0	0
External reflection	1L = 0	ITO Ref (default)	0.868	0	0
	2L = 0.023				
	3L = 0.046				
	4L = 0.070				
	5L = 0.092				
Surface condition	Plane	Plane	Plane	Plane	Plane
Internal reflection	0	0	0	0	0

**Table 2:** Parameters of the layers

Parameter	n-MoS_2_	a-Si:H(i)	p-cSi
Thickness	3L–6L (1L = 0.65 nm)	3–10 nm	50–500 μm
Dielectric constant (*ε*_r_)	4–14	11.9	11.9
Electron affinity *χ* (eV)	3.74–4.45	3.9	4.05
Band gap, *E*_g_ (eV)	1.41–1.48	1.6–2.0	1.12
Effective conduction band density, *N*_C_ (cm^−3^)	3 × 10^18^ to 9 × 10^20^	2.5 × 10^20^	3 × 10^19^ to 1 × 10^21^
Effective valence band density, *N*_V_ (cm^−3^)	3 × 10^18^ to 9 × 10^20^	2.5 × 10^20^	3 × 10^19^ to 1 × 10^21^
Effective electron mobility, *μ*_n_ (cm^2^ V^−1^ s^−1^)	517	20	1104
Effective hole mobility, *μ*_p_ (cm^2^ V^−1^ s^−1^)	8.5	5	420
Acceptor concentration *N*_A_ (cm^−3^)	0	0	10^15^ to 10^17^
Donor concentration *N*_D_ (cm^−3^)	10^12^ to 10^18^	0	0
Electron thermal velocity, *V*_e_ (cm s^−1^)	10^7^	10^7^	10^7^
Hole thermal velocity, *V*_h_ (cm s^−1^)	10^7^	10^7^	10^7^
Layer density, (g cm^−1^)	5.06	2.328	2.328
Auger electron recombination coefficient, (cm^6^ s^−1^)	∼10^−24^	0	2.2 × 10^−31^
Auger hole recombination coefficient, (cm^6^ s^−1^)	∼10^−24^	0	9.9 × 10^−32^
Band-to-band recombination coefficient, (cm^3^ s^−1^)	∼10^−7^	0	9.5 × 10^−15^
Defect type	Single	Conduction tail	Single
Defect charge	Acceptor	Acceptor	Acceptor
Total defect density (cm^−3^)	10^18^	6.4 × 10^19^	10^10^
Specific defect density (cm^−3^ eV^−1^)	10^18^	1.83 × 10^21^	10^10^
Defect level energy (eV)	0.6	0.035 (Urbach)	0.56
Electron and hole capture cross section (cm^−2^)	10^−14^ (default)	7 × 10^−16^	10^−14^
Optical properties	*n* = 4.47; *k* = 1.01	a-Si *nk* (default)	c-Si *nk* (default)

**Table 3:** Interfacial Parameters

Interface	Numerical model	Remarks
Graphene/n-MoS_2_	MS-Schottky contact	Surface recombination velocity for electrons and holes = 10^7^ cm s^−1^ (default)
p-cSi/Au		
n-MoS_2_/a-Si:H	Drift diffusion model	Interface charge = 10^11^ cm^−2^
a-Si:H/p-cSi		

### 2.3 Formation of the junctions

If n-MoS_2_ and p-cSi are brought into contact, a heterojunction forms and bending of the energy bands occur close to the Fermi level. This built-in potential (*V*_bi_) is developed at the MoS_2_/p-cSi interface. This voltage facilitates the separation of photo-generated carriers in the solar cell. Generally, in the p–n junction solar cell, the interfacial defect density reduces the overall efficiency of the cell. Therefore, better surface passivation between the two layers is required to reduce the defect density at the interface. Here, an intrinsic layer a-Si:H is inserted between n-MoS_2_ and p-cSi. This layer can be deposited on Si using the plasma-enhanced chemical vapor deposition method at a low temperature (<200 °C) without degrading the bulk properties of the Si substrate.^4^ Also, it introduces band offsets between the active and emitter band which are also beneficial to obstructing the reverse flow of the charge carriers. The schematic energy band diagram of the proposed HIT solar cell is shown in Fig. 1b. The build-in potential (*eV*_bi_) in the HIT solar cell is defined as:1
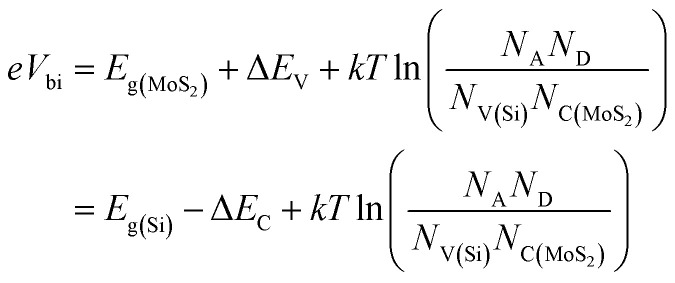
In which *e*, *E*_g_, Δ*E*_C_ and Δ*E*_V_, *k* and *T* are the electric charge, bandgap, the conduction band offset and valence band offset, Boltzmann constant, and the temperature in Kelvin, respectively. *N*_A_ and *N*_D_ represent the acceptor and donor concentrations and *N*_C_ and *N*_V_ are the conduction band and valence band density.^53^ In this HIT cell, a barrier for hole transportation is built at the interface between n-MoS_2_ and the pristine graphene corresponding to the potential energy of *eV*_bi_ which can be expressed using the following equation:^54^

2 *eV*_bi_ = *e*(*Φ*_MoS_2__ − *Φ*_Gr_)

In which, *Φ*_MoS_2__ and *Φ*_Gr_ are the work functions of n-MoS_2_ and pristine graphene respectively. As reported in previously published literature,^16*and*55^ the *Φ* of a six layer MoS_2_ (∼5.4 eV) is greater than that of pristine graphene (∼4.1 eV).

## 3. Results and discussion

### 3.1 Consequences of the n-MoS_2_ layer

**Fig. 2:**
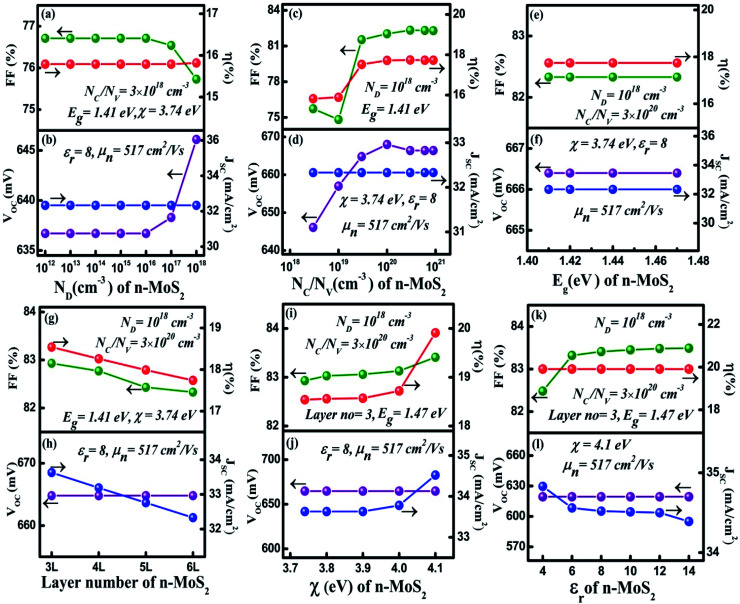
Optimization of the n-MoS_2_ layer: (a) and (b) donor concentration *N*_D_ (cm^−3^), (c) and (d) effective conduction band and valance band density *N*_C_/*N*_V_ (cm^−3^), (e) and (f) the bandgap energy *E*_g_ (eV), (g) and (h) the number of layers of MoS_2_, (i) and (j) the electron affinity *χ* (eV), and (k) and (l) the dielectric constant *ε*_r_. The p-cSi parameters were maintained as: thickness = 100 μm, *N*_A_ = 1 × 10^16^ cm^−3^, *N*_C_/*N*_V_ = 3 × 10^19^ cm^−3^ and *χ* = 4.05 eV, and the graphene parameters were maintained as: number of layers = 5, reflectance = 0.092 and work function = 4.31 eV. The other parameters are given in Tables 1 and 2.

In this section, we have optimized the donor *N*_D_ of the n-MoS_2_ layer in the range from 10^12^ to 10^18^ cm^−3^, and other parameters such as the thickness, *N*_C_/*N*_V_, *E*_g,_*χ* and *ε*_r_ are kept at feasible values as shown in Fig. 2a and b. In Fig. 2b, it can be seen that as *N*_D_ was increased from 10^12^ cm^−3^ to 10^16^ cm^−3^, the *V*_OC_ remained constant at the value of 636.7 mV. At *N*_D_ > 10^16^ cm^−3^, the *V*_OC_ increased to 646.1 mV as *N*_D_ was increased to 10^18^ cm^−3^. At a low value of *N*_D_, the consistency of *V*_OC_ indicates that it does not depend on *N*_D_ unless the condition Δ*n*_o_ ≫ *N*_D_ is followed, as reported previously,^56^ in which Δ*n*_o_ represents the excess electron–hole pairs. At *N*_D_ > 10^16^ cm^−3^, the *V*_OC_ increases owing to enhancement in the *eV*_bi_ according to eqn (2). As the mobility of the photo-generated carriers remains almost constant even with an increase of *N*_D_, this might keep *J*_SC_ constant at 32.33 mA cm^−2^. Similarly, the FF was found to be constant at 76.71% until the *N*_D_ reached 10^16^ cm^−3^ and then the FF slightly reduced to 75.73%. This is due to an increase in the recombination rate at a higher *N*_D_ (10^18^ cm^−3^). As we have observed, the *N*_D_ controls the *V*_OC_, thus the *η* of the cell remained constant at 15.79% as the *N*_D_ increases, and a maximum of *η* = 15.82% was obtained at *N*_D_ = 10^18^ cm^−3^.

The *N*_C_/*N*_V_ is related to the effective mass of the electrons 
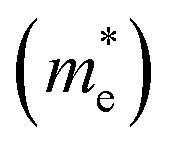
 and holes 
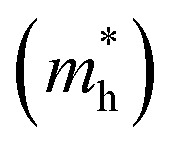
 as follows: 
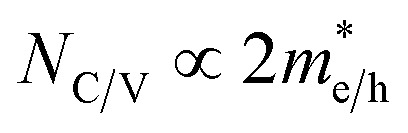
. Furthermore, 
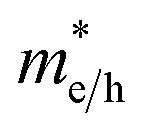
 is related to the mobility (*μ*_e/h_) as follows: 
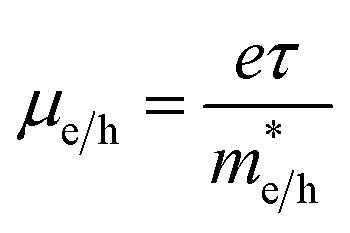
. From these two relationships, it can be concluded that with the change in mobility (*μ*_e/h_) the conduction and valence band density (*N*_C_/*N*_V_) also changes. It has been reported that the mobility of the charge carriers in MoS_2_ changes with the change in the number of layer. MoS_2_ has a mobility of 200 cm^2^ V^−1^ s^−1^ in the monolayer and approximately 517 cm^2^ V^−1^ s^−1^ in few layers.^11^ Therefore, after optimization of *N*_D_ to 10^18^ cm^−3^, the *N*_C_/*N*_V_ was optimized in the range 3 × 10^18^ to 9 × 10^20^ cm^−3^ as shown in Fig. 2c and d. It can be seen in Fig. 2d that the *V*_OC_ increased from 646.1 to 668 mV with an increase of the *N*_C_/*N*_V_ from 3 × 10^18^ cm^−3^ to 10^20^ cm^−3^. The increment in *V*_OC_ can be understood by considering the equation: 
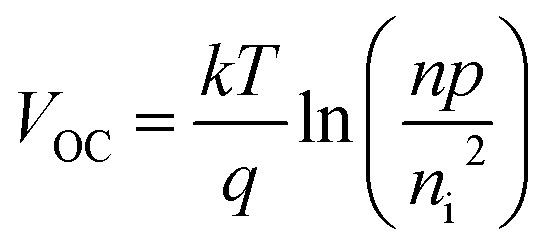
 in which *n*_i_ is the carrier concentration, *n* is the intrinsic electron concentration and *p* represents the intrinsic hole concentration. Here, *n* = *n*_o_ + Δ*n* and *p* = *p*_o_ + Δ*p*, in which *n*_o_ and *p*_o_ are the concentrations of the electrons and holes in the equilibrium condition and Δ*n*, Δ*p* are the excess photo-generated electron and hole concentrations, respectively. As, *n*_o_*p*_o_ = *n*_i_^2^, *n* = *N*_D_ and Δ*n* = Δ*p*, *V*_OC_ can therefore be rewritten as 
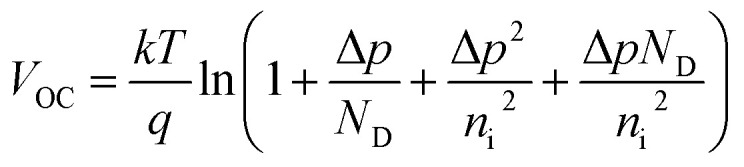
.^57^ We can assume that *V*_OC_ increased with *N*_C_/*N*_V_ owing to an increase of Δ*p* and Δ*n*. As shown in Fig. 2d, the *V*_OC_ decreased a little bit to 666.4 mV as the *N*_C_/*N*_V_ increased to 9 × 10^20^ cm^−3^, which is due to the decrement of *eV*_bi_ according to eqn (1). The *J*_SC_ was found to remain constant at 32.33 mA cm^−2^ with an increase of *N*_C_/*N*_V_ from 3 × 10^18^ to 9 × 10^20^ cm^−3^. Although the generation rate (*G*) of the photo-generated carriers is reported to increase with higher values of *N*_C_/*N*_V_.^58^ The constant *J*_SC_ indicates that the photo-generated carriers either recombine or are trapped close to the metal/semiconductor interface. As with the *V*_OC_, the FF is also found to increase and a maximum value of 82.33% was obtained at *N*_C_/*N*_V_ = 3 × 10^20^ cm^−3^. Therefore, *η* was found to be enhanced from 15.82% to a maximum value of 17.73%.

After optimization of the *N*_D_ and *N*_C_/*N*_V_ to values of 10^18^ cm^−3^ and 3 × 10^20^ cm^−3^, respectively we varied the bandgap (*E*_g_) in the range 1.41–1.47 eV (ref. 39) and results are shown in Fig. 2e and f. As we can see, all of the cell parameters are constant and a value of *η* = 17.74% was obtained in this range of *E*_g_.

For the simulation with different numbers of layers of n-MoS_2_ as shown in Fig. 2g and h, the *V*_OC_ was found to remain constant at a value of 664.8 mV, whereas the *J*_SC_ reduced from 33.63 to 32.33 mA cm^−2^, when the number of layers was increased from 3 to 6. This happens because the increase in the number of layers reduces the transmittance of photons to reach the junction, which results in a reduction of the electron–hole pair generation rate. The reduction of the transmittance with the increase in the n-MoS_2_ layer number leads to a reduction of the FF. The *η* was found to decrease from 18.54% to 17.74% as the number of layers increased from 3 to 6. Hence, we used 3L as the optimized number of layers of MoS_2_ for further simulation.

Fig. 2i and j show the cell parameters for the optimization of *χ* in the ranges 3.7 to 4.1 eV. Here we have considered *E*_g_ = 1.47 eV for 3L MoS_2_.^42^ The *V*_OC_ remained constant at a value of 664.8 mV when *χ* (eV) was increased from 3.7 to 4 eV. Upon a further increase in the *χ* to 4.1 eV, a small increase in the *V*_OC_ to approximately 691.4 eV was obtained owing to an increase in the *eV*_bi_. A constant *J*_SC_ of 33.63 mA cm^−2^ was found in the range 3.74–3.90 eV of *χ* and this was enhanced to 34.16 mA cm^−2^ for *χ* = 4.1 eV. A small increase in the Δ*E*_C_ (see Fig. 1b) was observed with the increase in *χ*, which leads to a slightly higher *V*_OC_ and *J*_SC_ at *χ* = 4.1 eV. The FF was observed to increase from 82.93% (@ *χ* = 3.74 eV) to 83.41% (@ *χ* = 4.1 eV) as a result of the reduction in the sheet resistance. Therefore, *η* was found to increase from 18.54% to 19.19%.

It has been found from electric transportation measurements that the dielectric response of the MoS_2_ layers is sensitive to the substrate that is used and the dielectric effect relies on the thickness of the sample.^43^ The effect of the dielectric constant (*ε*_r_) on the cell parameters is depicted in Fig. 2k and l. It can be observed in Fig. 2l, that the *V*_OC_ does not depend on the variation of *ε*_r_ and it was found to be a constant at 691.4 eV. However, the *J*_SC_ shows a light decrement from 34.83 to 34.39 mA cm^−2^ with an increase of *ε*_r_ up to 14. In a p–n junction, 
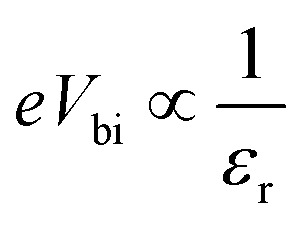
, which affect the relative permittivity of the MoS_2_ layer.^54,57^ The generation of the e–h pair increases in the MoS_2_ layer with an increase in the *ε*_r_ following Coulombs law as 
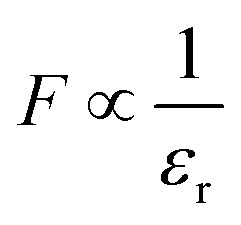
, in which *F* is the electrostatic force between the electron and holes, but at the same time the increase in *ε*_r_ reduces *eV*_bi_. As a consequence of both of these effects, we obtained a constant *V*_OC_. In contrast, under short circuit conditions, the photo-generated carriers experienced reduced *eV*_bi_, which boosts the recombination of the carriers. Thus, the *J*_SC_ was found to decrease. The FF showed a minor increase from 82.43% to 83.41% when *ε*_r_ was increased from 4 to 6. After this the increase in FF slowed down and only reached 83.49% for *ε*_r_ = 14. In Fig. 2k, *η* was found to largely depend on the *V*_OC_ and remained constant at 19.91% with *ε*_r_. The cell parameters of the proposed cell were found to be independent of the electron mobility (*μ*_n_) of the n-MoS_2_ layer, which has been varied in the range of 517–50 cm^2^ V^−1^ s^−1^.

### 3.2 Effect of the a-Si:H(i) layer thickness

**Fig. 3:**
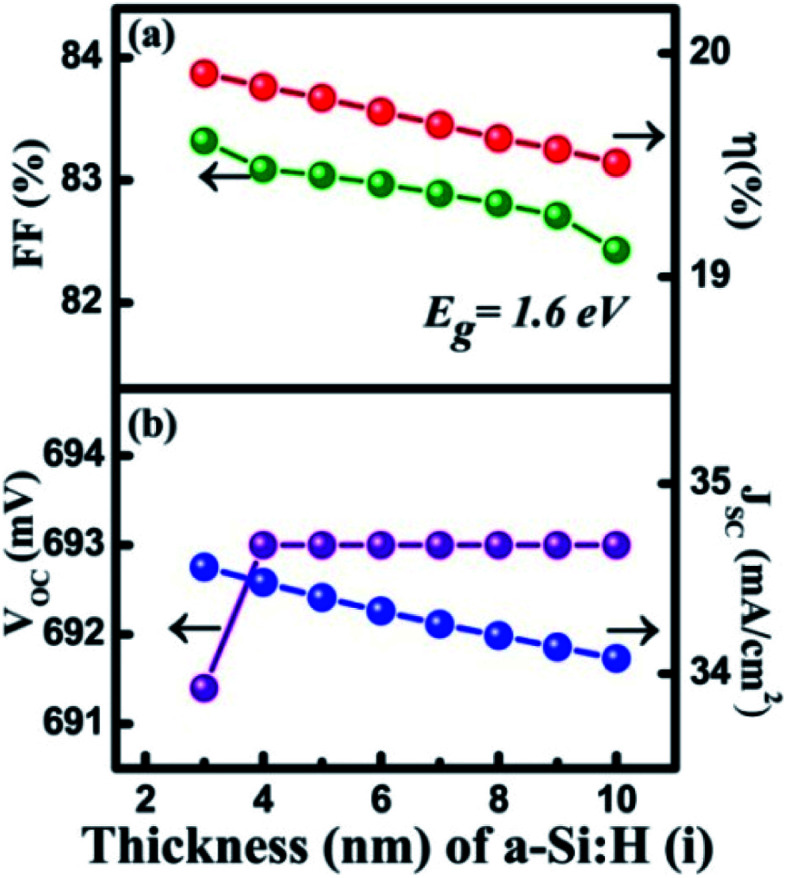
Optimization of the a-Si:H(i) layer: (a) and (b) thickness (nm) in which *E*_g_ was maintained at 1.6 eV.

In the HIT solar cell, an intrinsic layer of a-Si:H is inserted, mainly for passivation of the c-Si surface in the cell.^51^ Moreover, the insertion of the thin intrinsic layer also contributes to the depletion region, as well as to the drift current and electric field strength.^59^Fig. 3a and b shows the dependence of the cell parameters on the thickness variation of the a-Si:H(i) layer is in the 3 to 10 nm range. It can be observed in Fig. 3b that along with the increase of a-Si:H(i) thickness from 3 to 4 nm, the *V*_OC_ was slightly enhanced from 691.4 to 693 mV owing to a minor increase in the barrier height at the a-Si:H(i) layer and p-cSi interface.^43^ The *V*_OC_ then became constant at 693 mV upon further increasing the thickness of a-Si:H(i). The *J*_SC_ was found to decrease linearly from 34.56 to 34.08 mA cm^−2^. It is reported that a thicker intrinsic layer increases the series resistance in the cell.^50^ Therefore, we observed a tiny change in the *J*_SC._ Thus, the FF was found to reduce from 83.32% to 82.43%. Consequently, the *η* obtained reduced from 19.91% to 19.51%. To observe the cell performance for the optimized bandgap energy of a-Si:H(i), a further simulation was carried out by varying the *E*_g_ a-Si:H(i) in the range 1.6 to 2 eV. However, the maximum *η* = 19.19% was only obtained at *E*_g_ = 1.6 eV. This might be because the absorption of the photons by the MoS_2_ layer only corresponds to this *E*_g_ of the a-Si:H(i) layer.

### 3.3 Optimization of the p-cSi layer

**Fig. 4:**
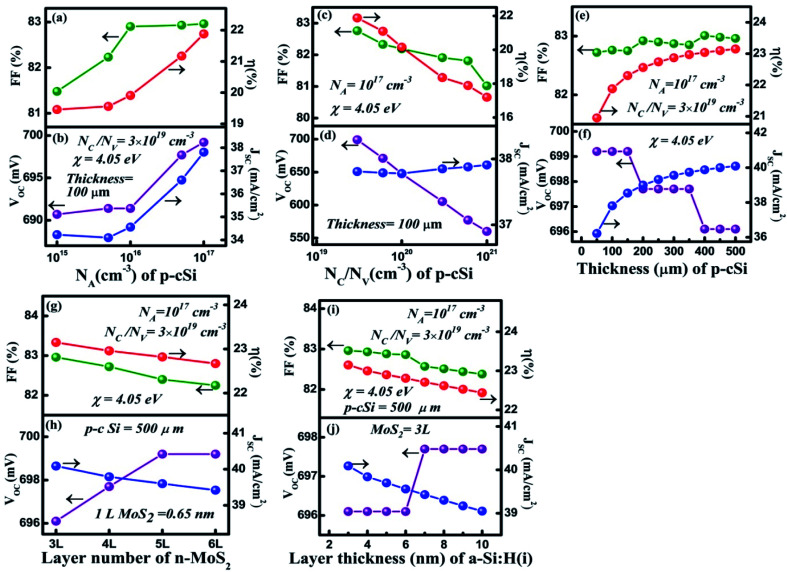
Optimization of the p-cSi layer: (a) and (b) acceptor concentration *N*_A_ (cm^−3^), (c) and (d) effective conduction band and valance band density *N*_C_/*N*_V_ (cm^−3^), (e) and (f) thickness (μm), (g) and (h) number of layers of MoS_2_ and (i) and (j) layer thickness of a-Si:H(i). The optimized parameters of a-Si:H(i) were maintained as: thickness = 3 nm, *E*_g_ = 1.6 eV, and the parameters for n-MoS_2_ were maintained as: *N*_D_ = 10^18^ cm^−3^, *N*_C_/*N*_V_ = 3 × 10^20^ cm^−3^, number of layers = 3, *E*_g_ = 1.47 eV, *χ* = 4.1 eV and *ε*_r_ = 6.

After optimizing the n-MoS_2_ and a-Si:H(i) parameters to their optimal values, the simulation was further performed to optimize the parameters of the p-cSi layer, while keeping the parameters of n-MoS_2_ and a-Si:H(i) at their optimal values, as discussed in the previous section. The results of the cell performance against variation in the *N*_A_ variation of p-cSi are shown in Fig. 4a and b. It is observed in Fig. 4b that when the *N*_A_ is increased from 10^15^ to 10^17^ cm^−3^, the *V*_OC_ also increased from 691.2 to 699.2 mV owing to the higher *eV*_bi_ with *N*_A_ according to eqn (1). Similarly, *J*_SC_ was also found to increase from 34.23 to 39.15 mA cm^−2^ as the increased *eV*_*b*_ pushes the photo-generated carriers effectively from the depletion region into the contact region without experiencing a recombination effect or the charge carrier trapped effect in the neutral region. This resulted in an increment in the *J*_SC_.^57^ The increment in the FF from 81.48% to 82.76% can be attributed to the reduction of the sheet resistance as *J*_SC_ is also increased. These changes lead to an improvement in *η* from 19.46% to 21.88%. Hence, *N*_A_ = 10^17^ cm^−3^ with a maximum *η* of 21.88% is considered to be the optimal value for the c-Si layer.

To understand the effect of *N*_C_ and *N*_V_ on the performance of the HIT solar cell, we varied these parameters within the range of 3 × 10^19^ to 1 × 10^21^ cm^−3^ for the p-cSi layer. It can be observed from Fig. 4c and d, with the increase in the *N*_C_/*N*_V_, the *V*_OC_ decreased linearly from 699.2 to 560.2 mV. According to eqn (1), with the increase in the *N*_V_ of p-cSi layer the *eV*_bi_ decreases and therefore *V*_OC_ also decreases. In contrast, the value of *J*_SC_ shows a small increase from 37.81 to 37.91 mA cm^−2^ with *N*_C_/*N*_V_. It has also been reported that *N*_C_/*N*_V_ influences the bandgap as well as the absorption of photons.^58^ It can be estimated that for *N*_C_/*N*_V_ > 3 × 10^19^ cm^−3^, the high absorption of photons in the active silicon layer increased the *G* of electron–hole pairs. In Fig. 4d, the FF showed a deterioration from 82.76% to 81.02%, which resulted in the *η* dropping to 17.2% from 21.88%. The maximum value of *η* = 21.88% was obtained at *N*_C_/*N*_V_ = 3 × 10^19^ cm^−3^.

In solar cell technology, the thickness of the p-cSi wafer plays an important role in the fabrication of HIT solar cells. Fig. 4e and f represents the variation of cell parameters as a function of the p-cSi wafer thickness from 50 to 500 μm. In Fig. 4f, the *V*_OC_ remains constant at 699.2 mV. Upon a further increase in the thickness, the *V*_OC_ reduced sharply to 697.7 mV and again remained constant up to a thickness of 350 μm. Above this thickness, the *V*_OC_ was found to deteriorate to the value 696.1 mV and becomes independent of the change of thickness. This variation may be due to the variation in the recombination of the photo-generated carriers under open circuit conditions. The observed enhancement in the *J*_SC_ from 36.22 to 40.09 mA cm^−2^ can be ascribed to the boost in the generation of the charge carriers with the increase in the p-cSi thickness under short circuit conditions. The FF was observed to increase slowly from 82.72% to 82.96% owing to the reduction in the series resistance. These processes help to increase the *η* from 20.95% to 23.15% at a thickness of 500 μm.

To study the role of the number of layers of n-MoS_2_ and the a-Si:H(i) layer thickness with the best optimized p-cSi parameters, the proposed cell was again simulated by varying the number of layers of MoS_2_ and the thickness of a-Si:H and the results are depicted in Fig. 4g, h and i, j respectively. From Fig. 4g and h, it can be clearly seen that the *V*_OC_ increased from 696.1 to 699.2 mV when the number of layers of n-MoS_2_ was increased from 3 to 5. Furthermore, the increase in the number of layers to 6*V*_OC_ remains constant at 699.2 mV. On the other hand, the *J*_SC_, FF, and *η* were observed to decrease with the increase in the number of layers. The *J*_SC_ decreased from 40.09% to 39.42%, the FF from 82.96% to 82.25 and the *η* from 23.15% to 22.67%. In Fig. 4i and j, as the layer thickness of a-Si:H(i) was changed to 6 from 3 nm, the *V*_OC_ gave a constant value of 696.1 mV and increased slightly to 697.7 mV when the thickness was increased to 6 nm. The *V*_OC_ then became independent of the thickness even when it was increased to 10 nm. The *J*_SC_ was found to decline from 40.09 to 39.05 mA cm^−2^ with the thickness of a-Si:H(i). The FF was found to decrease from 82.96% to 82.38% and a minimum *η* of 22.14% was achieved at 10 nm.

### 3.4 Simulation and optimization of the front contact (graphene) parameters

**Fig. 5:**
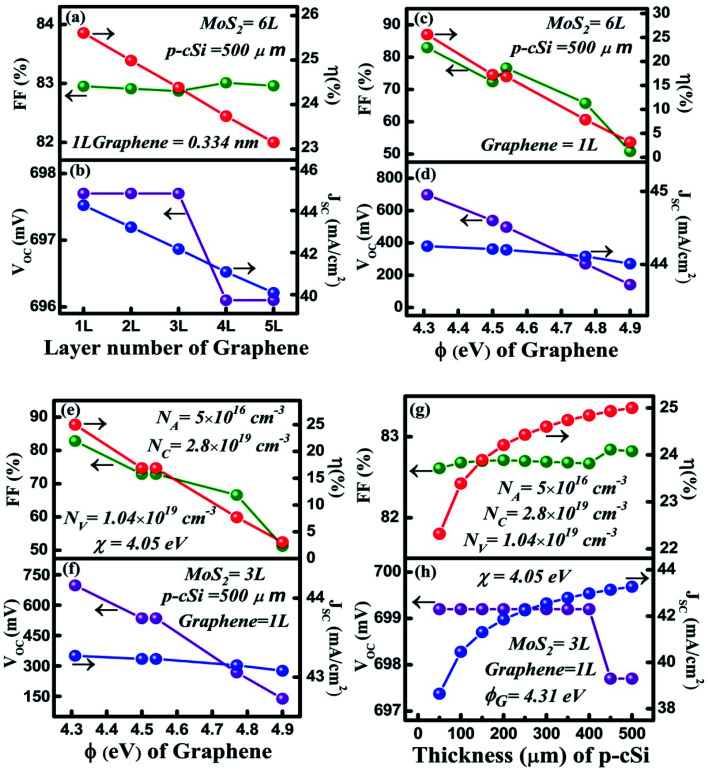
Optimization of the graphene (front contact) layer: (a) and (b) optimization of the graphene layer number, (c) and (d) optimization of the *Φ* (eV) of the graphene layer in which the parameters of n-MoS_2_ and p-cSi are maintained at their best-optimized values, (e) and (f) optimization of the *Φ* (eV) of the graphene layer and (g) and (h) optimization of the thickness of a commercially available silicon wafer with practically available p-cSi parameters.

In a solar cell, contact plays a transparent conducting electrode role in the solar cell by allowing the light to transmit through and maintain a high electrical conductivity. Fig. 5a and b shows the dependence of the HIT solar cell parameters on the variation of the graphene layer (front contact) number. It is observed in Fig. 5b that with the increase in the number of graphene layers from 1 to 3, the *V*_OC_ remained constant at 697.7 mV. The reason behind this consistency might be the decrease in the recombination rate which limits the reduction of the charge carrier generation under open-circuit voltage conditions. However, upon further increasing of the graphene layer number to 4, the absorbed light might not be sufficient to produce an efficient electron–hole pair, which causes the *V*_OC_ to slightly decrease to 696.5 mV and then become constant with an increase in the layer number. On the other hand, the *J*_SC_ was observed to decline linearly from 44.25 to 40.09 mA cm^−2^. When the number of layers of graphene was increased the optical transmittance reduced and thus the *G* of the charge carriers decreased and therefore the *J*_SC_ reduced too. Under short circuit conditions, the photo-generated carriers could be trapped close to the graphene/n-MoS_2_ junction. Therefore, we have observed a linear decrease in the *J*_SC_, which also, in turn, reduced the *η* from 25.61% to 23.15%. An insignificant change in the FF was found from 82.95% to 82.96%.

The presence of a strong covalent bond between the C and Mo atoms or the presence of a dangling bond of sulfur present in the interfaces affects the interfacial barrier height and a variation in the barrier height results in a variation in the contact resistance.^60,61^ Usually, the contact resistance is a vital factor that affects the solar cell performance.^62^ However, many experimental reports suggest that by controlling the Fermi level (*i.e.* the work function) of graphene, the contact resistance can be minimized in the graphene/MoS_2_ interfaces.^63,64^ Therefore, in this work, we have focused primarily on the work function of graphene. The depicted results shown in Fig. 5c and d, show the variation of the cell parameters with the variation of the graphene work function (*Φ*_Gr_) from 4.31 to 4.9 eV. A large reduction in the *V*_OC_ was observed in Fig. 5d, in which it decreased from 697.7 to 141.4 mV with an increase of *Φ*_Gr_. From eqn (2), it can be observed that with the increase in *Φ*_Gr_, the potential barrier energy *eV*_bi_ of graphene/n-MoS_2_ decreases. It has also been reported that when the *Φ*_TCO_ (contact) of Si HIT solar is low, the *eV*_bi_ of the TCO (transparent conducting oxide)/n-type emitter has the same direction as that of the *eV*_bi_ of the n-emitter/p-Si. On the other hand, when the *Φ*_TCO_ (contact) is high, the *eV*_bi_ of the TCO/n-type emitter contact will have an inverted direction to that of the *eV*_bi_ of the n-emitter/p-Si junction. Moreover, with the increase in the *Φ* of the TCO contact in the silicon HIT solar cell, the depletion region in the emitter region increases and widens and results in the overlap of the contact/n-type emitter region and the n-type emitter/p-type silicon junction region if the emitter is not sufficiently thin enough.^4^ Thus, the higher *Φ*_Gr_ might increase the possibility of overlapping between the graphene/n-MoS_2_ depletion region and the n-MoS_2_/p-cSi depletion region, which has resulted in a reduction in the *V*_OC_. In contrast, owing to the overlap of the graphene/n-MoS_2_ depletion region and the n-MoS_2_/p-cSi depletion region, the *eV*_bi_ of n-MoS_2_/p-cSi reduces. This results in a reduction of the built-in electric field which is unable to push the photo-generated carriers effectively to their respective sides under short circuit conditions and thus the *J*_SC_ is observed to decline linearly from 44.25 to 40.09 mA cm^−2^ with an increase in the *Φ*_Gr_. The overall reduction in the *V*_OC_ and *J*_SC_ reduced *η* from 25.61% to 23.15%. The maximum *η* = 25.61% was achieved at *Φ*_Gr_ = 4.31 eV of pristine graphene.

To validate our simulated results, a comparison has been performed by considering the best-optimized values of n-MoS_2_ and a-Si:H(i) with the practically available p-cSi parameters by varying the *Φ*_Gr_ and p-cSi thickness within the range of 4.31–4.9 eV and the 50–500 μm range respectively, as depicted in Fig. 5e–h and Table 4, using the practically available data for p-cSi.^52,65^ It can be seen from Fig. 5e and f, that the maximum *η* = 25% was obtained at *Φ*_Gr_ = 4.31 eV, which is close to our best-optimized results as shown in Fig. 5c and d. Similarly, in Fig. 5g and h, the same maximum *η* = 25% was achieved for a 500 μm thick p-cSi wafer.

**Table 4:** Summary of the most optimized cell

Cell parameter	Most optimized cell (before optimization of the parameters of the graphene layers)	Graphene layer number optimized cell (after optimization of n-MoS_2_ and p-cSi)	n-cSi wafer optimized cell for practically available silicon parameters
*V*_OC_ (mV)	696.1	697.7	697.7
*J*_SC_ (mA cm^−2^)	40.09	44.25	43.27
FF (%)	82.96	82.95	82.82
*η* (%)	23.15 [@ 3L n-MoS_2_ at *N*_D_ = 10^18^ cm^−3^, *N*_C_/*N*_V_ = 3 × 10^20^ cm^−3^, *E*_g_ = 1.47 eV, *χ* = 4.47 eV, *ε*_r_ = 6, *μ*_n_ = 517 cm^2^ V^−1^ s^−1^, *μ*_p_ = 8.5 cm^2^ V^−1^ s^−1^, and @ 6 nm a-Si:H(i) at *E*_g_ = 1.6 eV and @ 500 μm p-cSi at *N*_A_ = 1 × 10^17^ cm^−3^, *N*_C_/*N*_V_ = 3 × 10^19^ cm^−3^, *χ* = 4.05 eV and @ 5L graphene at *Φ* = 4.31 eV]	25.16 [@ 3L n-MoS_2_ at *N*_D_ = 10^18^ cm^−3^, *N*_C_/*N*_V_ = 3 × 10^20^ cm^−3^, *E*_g_ = 1.47 eV, *χ* = 4.47 eV, *ε*_r_ = 6, *μ*_n_ = 517 cm^2^ V^−1^ s^−1^, *μ*_p_ = 8.5 cm^2^ V^−1^ s^−1^, and @ 6 nm a-Si:H(i) at *E*_g_ = 1.6 eV and @ 500 μm p-cSi at *N*_A_ = 1 × 10^17^ cm^−3^, *N*_C_/*N*_V_ = 3 × 10^19^ cm^−3^, *χ* = 4.05 eV and @ 1L graphene at *Φ* = 4.31 eV]	25 [@ 3L n-MoS_2_ at *N*_D_ = 10^18^ cm^−3^, *N*_C_/*N*_V_ = 3 × 10^20^ cm^−3^, *E*_g_ = 1.47 eV, *χ* = 4.47 eV, *ε*_r_ = 6, *μ*_n_ = 517 cm^2^ V^−1^ s^−1^, *μ*_p_ = 8.5 cm^2^ V^−1^ s^−1^, and @ 6 nm a-Si:H(i) at *E*_g_ = 1.6 eV and @ 500 μm p-cSi at *N*_A_ = 5 × 10^16^ cm^−3^, *N*_C_/*N*_V_ = 2.8 × 10^19^/1.04 × 10^19^ cm^−3^, *χ* = 4.05 eV and @ 1L graphene at *Φ* = 4.31 eV]

### 3.5 Variation and simulation of the front contact and back contact material

**Fig. 6:**
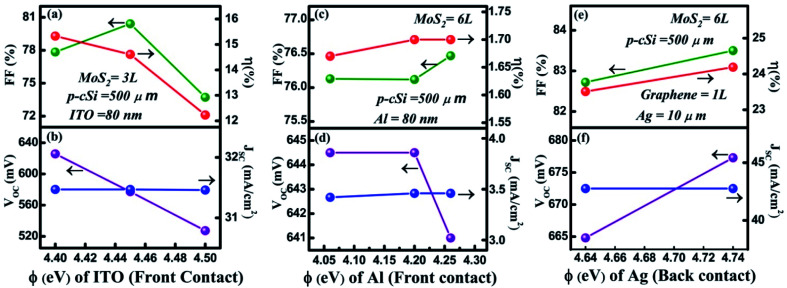
Changing the front and back contact: (a) and (b) variation in the *Φ* (eV) of ITO, (c) and (d) variation in the *Φ* (eV) of Al, and (e) and (f) variation in the *Φ* (eV) of Ag.

In this section, a comparison of the cell parameters has been performed by changing the front contact material graphene to ITO and Al^19^ and the back contact to Ag. As seen in Fig. 6a and b the thickness of ITO is considered to be 80 nm and the *Φ*_ITO_ has been varied from 4.40–4.50 eV.^34^ The maximum values for *V*_OC_, *J*_SC_, FF and *η* were obtained and found to be 625.8 mV, 31.47 mA cm^−2^, 80.41% and 15.33% respectively. Similarly, in Fig. 6c and d, when *Φ*_Al_ was varied, the maximum values for *V*_OC_, *J*_SC_, FF, and *η* were found to be 650.8 mV, 4.36 mA cm^−2^, 82.79% and 2.353%, respectively. In both cases, *η* was significantly lower than that of *η* obtained using the graphene layer as a contact.

Fig. 6e and f shows the variation in the cell parameters when the back contact was changed from Au to Ag and the *Φ* of Ag was maintained from 4.64 to 4.74 eV. It was observed that in this range of *Φ* for Ag, the *V*_OC_ increased from 664.8 to 677.3 mV, whereas the *J*_SC_ remained constant at 42.76 mA cm^−2^. The FF and *η* were observed to decrease from 82.72% to 83.5% and 23.51% to 24.19%, respectively. This *η* was close to our simulated *η*_max_ value, that is, 25.61% when we used Au as a contact. Here, the back contacts have been considered as the uniform thin film deposited on the backside of p-cSi.

### 3.6 maximum power and series resistance

**Fig. 7:**
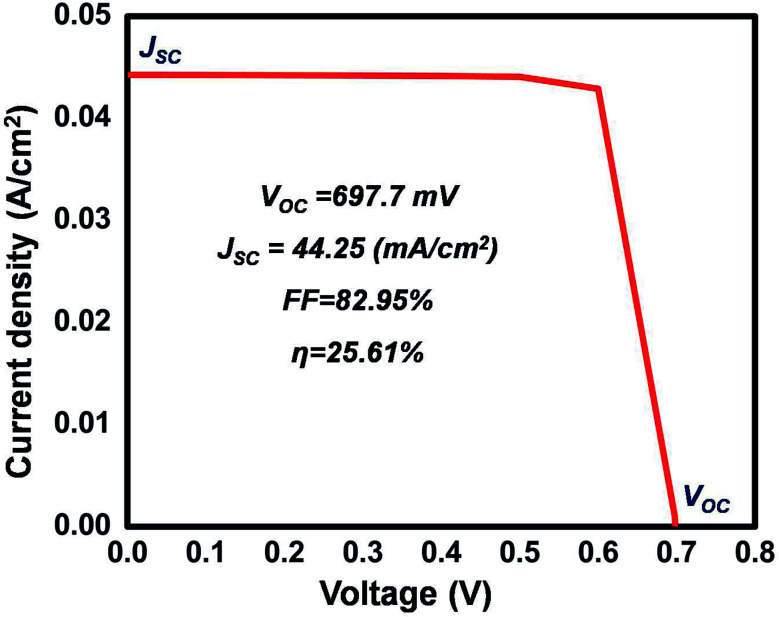
*IV* characteristics of the most optimized cell.

The proposed solar cell as shown in Fig. 1a was simulated and characterized under a standard AM1.5G spectrum and forward voltage in the range of 0 to 0.7 V. The *IV* characteristics of the best optimized solar cell generated by the software are shown in Fig. 7. The maximum power delivered by the cell, as well as the series resistance (*R*_s_), was calculated from the *IV* characteristics and found to be 25.4 mW cm^−2^ and 2.5 Ω cm^2^ was achieved respectively (see ESI†).

Finally, a comparison between the experimental and theoretical demonstration of graphene, MoS_2,_ and Si-based solar cells was performed and the results are detailed in Table 5. However, except for our work none of the studies used graphene as a contact material or TCE.

**Table 5:** Graphene and MoS_2_ silicon-based solar cells

Type of study	Solar cell structure	*V*_OC_ (V)	*J*_SC_ (mA cm^−2^)	FF (%)	*η* (%)	Ref.
Experimental	ITO/graphene/MoS_2_/n-cSi/(Ti/Ag)	0.59	36.8	73	15.8	66
Experimental	Au/graphene/MoS_2_/n-Si/In	0.56	33.4	60	11.1	67
Experimental	Au/graphene/MoS_2_/n-Si/Au	0.50	28.1	47	6.56	68
Experimental	Pd/n-MoS_2_/i-SiO_2_/p-Si/In	0.30	5.5	42	4.5	69
Experimental	Ni/graphene/MoS_2_/p-csi/Al	0.51	—	—	2.58	70
Theoretical	TCO/MoS_2_/SiGe:H/p-Si/Al	0.652	40.01	83.7	21.85	3
Theoretical	Graphene/n-MoS_2_/a-Si:H/p-cSi/Au	0.697	44.25	82.95	25.61	This work

## 4. Conclusions

In order to develop an efficient HIT solar cell, a graphene/n-MoS_2_/a-Si:H(i)/p-cSi/Au structure has been proposed and simulated using AFORS-HET v 2.5 software. The individual effects of the six parameters of the n-MoS_2_ layer were analyzed individually. After optimizing the parameters of the n-MoS_2_, a-Si:H(i), and p-cSi layers, an optimum efficiency of 23.15% was achieved. As the contact material in a solar cell has an adverse effect on the cell performance, a further simulation was performed to observe the effect of graphene on the proposed HIT solar cell by varying the number of layers and the work function. From this simulation, a maximum efficiency of 25.61% was achieved for single-layer graphene at a work function of 4.1 eV. On the other hand, by changing the front contact graphene to ITO and Al we achieved efficiencies of 15.33% and 2.35%. This confirms that graphene could be an efficient contact material for use in an n-MoS_2_/a-Si:H(i)/p-cSi/Au HIT solar cell. Our simulated results establish that the graphene layer could be an effective contact TCE material and n-MoS_2_ could be an efficient emitter layer in a single facial n-MoS_2_ and p-cSi based HIT solar cell.

## Conflicts of interest

There are no conflicts to declare.

## Supplementary Material

NA-002-D0NA00309C-s001
