# Supplementary material for: The prospective application of a graphene/MoS2 heterostructure in Si-HIT solar cells for higher efficiency
Source: Nanoscale Adv. 2020 Jun 23;2(8):3231–43. doi: 10.1039/d0na00309c (PMC9418949; doi:10.1039/d0na00309c)
Supplement: NA-002-D0NA00309C-s001 [file NA-002-D0NA00309C-s001.pdf]

Electronic Supplementary Material (ESI) for Nanoscale Advances.
This journal is © The Royal Society of Chemistry 2020

Supplementary Information for

Prospective Application of Graphene/ MoS_2 Heterostructure in Si-HIT solar cell for Higher Efficiency

Chandra Kamal Borah[1], Pawan K. Tyagi[2] and Sanjeev Kumar[1*]

[1]Centre of Advanced Research, Department of Physics, Rajiv Gandhi University, Arunachal Pradesh-791112, India

[2] Department of Physics, Central University of Haryana, Haryana-123029, India

S.1: Calculation of maximum power and series resistance:

The series (R_s) of 2.5Ω.cm^2 was achieved, using a simple method as prescribed in the previous literature. This value is compatible with the value (8.8 Ω.cm^2-30 Ωcm^2) of the experimentally demonstrated graphene/MoS_2/silicon-based solar cell [2]. Likewise, a maximum power (P_{max}) of 25.4 mW/cm^2 was achieved using the formula as described in ref.3.

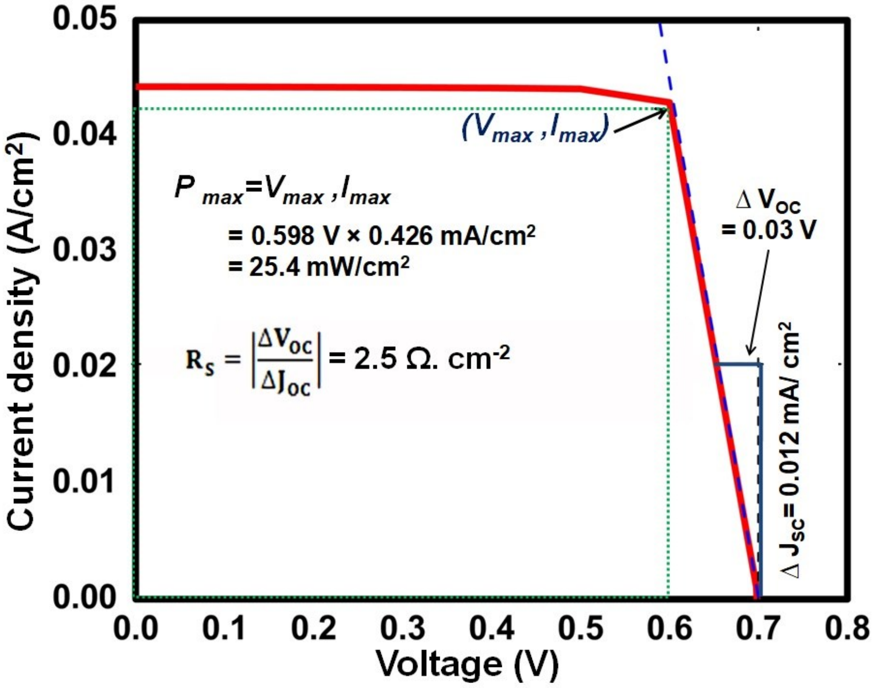

Fig. S1: Calculation of maximum power (P_{max}) and series resistance (R_s) of the best optimized cell

*Corresponding author:
E-mail addresses: sanjeev.kumar@rgu.ac.in

References:

1. M. Diantoro, T. Suprayogi, A. Hidayat, A. Taufiq, A. Fuad, and R. Suryana, *International Journal of Photoenergy*, 2018, https://doi.org/10.1155/2018/9214820

2. Y. Tsuboi, F. Wang, D. Kozawa, K. Funahashi, S. Mouri, Y. Miyauchi, T. Takenobub and K. Matsuda, *Nanoscale*, 2015,**7**, 14476-14482

3. J. Cubas , S. Pindado and C. de Manuel, Conference Proceedings Paper – Energies 2014 DOI: 10.3390/ece-1-c013
